# Complete Mitochondrial Genome and Phylogenetic Analysis of *Turdus pallidus* (Passeriformes, Turdidae)

**DOI:** 10.3390/genes16111284

**Published:** 2025-10-29

**Authors:** Qingbin Zhan, Yin Tang, Xiaoyan Zhao, Xiaoming Xue, Yunxia Chen, Yalin Huang

**Affiliations:** 1 Department of Criminal Science and Technology, Nanjing Police University, Nanjing 210023, China; zhanqb@njpu.edu.cn (Q.Z.); tangy@njpu.edu.cn (Y.T.); xuexm@njpu.edu.cn (X.X.); chenyx@njpu.edu.cn (Y.C.); 2 Key Laboratory of State Forestry and Grassland Administration on Wildlife Evidence Technology, Nanjing 210023, China; 3 School of Grassland Science, Beijing Forestry University, Beijing 100083, China; zhao0223@bjfu.edu.cn

**Keywords:** *Turdus pallidus*, mitochondrial genome, codon usage bias, phylogenetic analysis, avian evolution

## Abstract

Background: Thrushes (family Turdidae) are ecologically important passerine birds widely distributed across the Northern Hemisphere. However, the phylogenetic placement of several East Asian congeners, including *Turdus pallidus*, remains insufficiently resolved due to the limited resolution of partial mitochondrial or nuclear markers used in previous studies. Methods: In this work, we sequenced and annotated the complete mitochondrial genome of *T. pallidus* (16,739 bp) using high-throughput Illumina sequencing. The mitogenome exhibited the typical circular architecture and contained 37 genes (13 protein-coding genes, 22 tRNAs, and 2 rRNAs), with an overall GC content of 47.32%. Results: Most protein-coding genes initiated with the standard ATG codon, although lineage-specific deviations such as GTG in COX1 and ND2 were identified, and incomplete stop codons (T– or TA–) were observed, consistent with post-transcriptional polyadenylation. The 22 tRNA genes displayed typical cloverleaf secondary structures, except for trnS(AGN), which lacked a DHU arm, while rRNA genes were 977 bp (12S, 48.52% GC) and 1590 bp (16S, 44.65% GC), showing conserved stem regions but variable loop regions. Codon usage analysis revealed a strong bias toward A/T-ending codons, with a total of 3798 codons and an effective number of codons (ENC) of ~40, indicating moderate codon bias shaped by both mutational pressure and translational selection. Comparative analysis of evolutionary rates demonstrated that conserved genes such as COX1 and CYTB are suitable for resolving deeper relationships, whereas rapidly evolving genes like ATP8 provide resolution among closely related taxa. Conclusions: Phylogenetic reconstructions based on 13 mitochondrial protein-coding genes robustly supported the monophyly of Turdidae and recovered *T. pallidus* as most closely related to *T. obscurus*. Overall, this study provides a novel mitogenomic resource for *T. pallidus*, enhances phylogenetic resolution within *Turdus*, and underscores the value of complete mitochondrial genomes for molecular identification, conservation management, and avian evolutionary studies.

## 1. Introduction

Thrushes (family Turdidae) are a diverse and adaptable group of passerine birds found across most of the world, especially in Eurasia, Africa, and the Americas [1]. They play important roles in ecosystems as seed dispersers and insect predators, helping maintain ecological balance and biodiversity [2]. Because of their wide distribution, ecological flexibility, and appeal to birdwatchers, thrushes are valuable subjects for studies in ecology, evolution, and conservation [3,4,5]. The genus *Turdus* is one of the largest in Turdidae, with more than 60 species that live in a variety of habitats, from dense forests to open grasslands and cities [3,6,7]. This ecological and morphological diversity makes *Turdus* an important group for studying adaptation, niche use, and the formation of biodiversity. Phylogeographic studies have shown that mountains, oceans, and other barriers, together with past climate changes, have shaped the genetic structure and distribution of these birds. These events likely influenced their diversification and current patterns of occurrence [1,3,8,9].

The *Turdus pallidus* is a representative species in these studies. Early research on its relationships with other East Asian species in the genus used mitochondrial genes (e.g., ND2, ND3, CYTB) and a few nuclear genes (e.g., C-MOS). These studies generally placed *T. pallidus* close to several related *Turdus* species, but the short length and limited information of these sequences reduced the accuracy of the results [10,11,12,13]. The development of high-throughput sequencing now allows the use of complete mitochondrial genomes, which provide much more genetic information and improve the accuracy of phylogenetic analyses [14]. The mitochondrial genome is a small circular double-stranded DNA molecule of about 16–23 kb, containing 13 protein-coding genes, 22 tRNAs, 2 rRNAs, and a non-coding control region (D-loop) [15]. It typically shows an AT-rich base composition and a conserved gene order among closely related species. These features make the complete mitochondrial genome a powerful tool for species identification, phylogenetic studies, population genetics, and research on adaptive evolution and historical biogeography [16].

Based on this, we sequenced and annotated the complete mitochondrial genome of Pale Thrush *T. pallidus* using samples provided by the Pinghu Public Security Bureau, Zhejiang, China. We systematically analyzed its gene composition, gene order, nucleotide composition, and coding characteristics, and constructed a passerine phylogenetic tree using available database sequences to clarify the phylogenetic position of *T. pallidus* within the genus *Turdus*. This study provides reference data for molecular and forensic species identification, and offers an important theoretical basis and data support for the conservation management of *T. pallidus* and research on avian evolution.

## 2. Materials and Methods

### 2.1. Sample Collection

Muscle tissue of *T. pallidus* was provided by the Pinghu Public Security Bureau for forensic examination. It was from an illegal hunting case in Pinghu City (30.6696° N, 121.0221° E) in February 2024. Following Sangster & Luksenburg (2021) [17], we verified the identity of our mitogenome sequence of *T. pallidus* with reference sequences of three commonly used markers in songbird systematics: NADH dehydrogenase subunit 2 (*ND2*, 1041 bp; *n* = 2785, incl. one of *T. pallidus*), part of cytochrome c oxidase subunit I (*COX1*, 696 bp; *n* = 2610, incl. 13 of *T. pallidus*), and cytochrome *b* (*CYTB*, 1143 bp; *n* = 1140, incl. three of *T. pallidus*). In each of these analyses, our sequence of *T. pallidus* clustered with the reference sequences of *T. pallidus*, indicating that our sample was correctly identified. Samples were immediately stored at −20 °C until processing. Reagents included lysis buffer, proteinase K, phenol, chloroform, isoamyl alcohol, absolute ethanol, 70% (*v*/*v*) ethanol, and TE buffer. Major equipment comprised a high-speed microcentrifuge, pipettes, a temperature-controlled water/metal bath, a UV spectrophotometer (NanoDrop, Thalassery, India), and standard agarose gel electrophoresis and imaging systems.

### 2.2. Methods

#### 2.2.1. Genomic DNA Extraction and Quality Control

Approximately 30 mg of muscle tissue was homogenized in lysis buffer using a glass homogenizer, followed by overnight digestion with proteinase K at 55 °C. DNA was extracted with a phenol–chloroform–isoamyl alcohol mixture and centrifuged at 12,000 rpm for 10 min at 4 °C. The aqueous phase was collected; DNA was precipitated with absolute ethanol, washed with 70% ethanol, air-dried at room temperature, and resuspended in TE buffer. DNA concentration and purity were assessed with a NanoDrop spectrophotometer, and integrity was evaluated by 1% agarose gel electrophoresis. Qualified DNA (accession number: DNA-2024-54) was labeled and stored at −20 °C. Approximately 100 ng of DNA was used for sequencing, and the final sequencing volume was 30 μL per sample.

#### 2.2.2. High-Throughput Sequencing and Genome Assembly

Approximately 2 µg of qualified DNA libraries was sequenced on an Illumina HiSeq 2500 platform (Illumina，San Diego, CA , USA) using a paired-end strategy with 150-bp reads. Raw reads were assessed with FastQC v0.11.9 and trimmed with Trimmomatic v0.32 to remove adapters and low-quality bases [18,19]. De novo assembly was performed with SPAdes 3.15.2 [20]. Mitochondrial contigs were identified by BLAST 2.17.0 searches against avian mitogenomes available in NCBI. Manual joining and curation were conducted in Geneious Prime 2020.2 to obtain a complete circular mitochondrial genome [21]. Unless otherwise stated, default parameters were used.

#### 2.2.3. Annotation and Visualization

Gene prediction and preliminary annotation were carried out with the GeSeq web server [22], and protein-coding genes were confirmed by comparison with NCBI RefSeq mitochondrial proteins. The locations and secondary structures of tRNA and rRNA genes were predicted using ARAGORN v2.36 and tRNAscan-SE 2.0, respectively [23,24], and annotations were verified by BLAST searches against the NCBI nr database. Additional annotation support was obtained from MITOS2, followed by manual curation based on closely related species to generate a standardized tbl file [25]. Circular genome maps and structural visualizations were produced in CGView 1.0 [26].

#### 2.2.4. Phylogenetic Analysis

Thirteen protein-coding genes (PCGs) from the complete mitochondrial genome of *T. pallidus* were extracted and combined with homologous sequences from related passerine species retrieved from the NCBI GenBank database, including multiple *Turdus* congeners and other closely related taxa. Each PCG was aligned at the amino acid using MAFFT v7 [27], and ambiguously aligned regions were removed with Gblocks v0.91b [28]. The cleaned alignments were concatenated into a single dataset. The best-fit substitution models for each partition were determined with PartitionFinder v 2.1.1 [29], and selected models included GTR + I + G for all partitions. Maximum-likelihood (ML) analyses were performed in IQ-TREE v2 with 1000 ultrafast bootstrap replicates to evaluate nodal support. Resulting phylogenetic trees were visualized and edited in FigTree v1.4.4 [30].

## 3. Results

### 3.1. General Features and Protein-Coding Gene Organization of the T. pallidus Mitochondrial Genome

**Figure 1:**
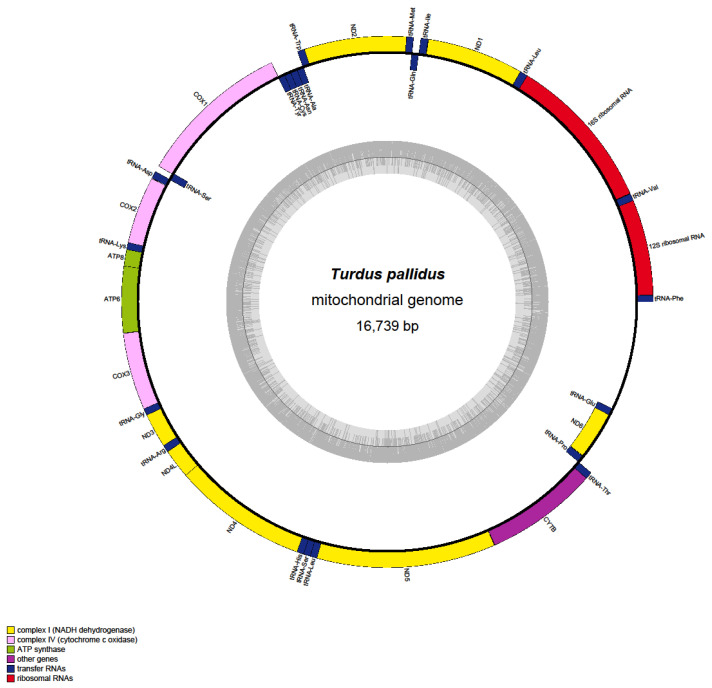
Circular map of the *T. pallidus* mitogenome, showing the arrangement of 37 genes.

The complete mitochondrial genome of *T. pallidus* (GenBank accession: PQ249424) was determined to be 16,739 bp in length, showing the typical circular structure conserved in avian species (Figure 1). The overall GC content was 47.32%, with A = 28.45%, C = 30.97%, G = 16.35%, and T = 24.23% (Table S1). The 13 PCGs collectively span 11,398 bp of the genome. Among them, the longest gene is ND5 (1818 bp), whereas the shortest is ATP8 (168 bp). Most PCGs initiate with the standard start codon ATG, but deviations were identified in COX1 and ND2, both of which initiate with the non-canonical codon GTG. Stop codon usage also exhibited variability: while most PCGs terminate with canonical TAA or TAG, incomplete termination codons (single T or TA–) were observed, such as in ND4, ND2, and COX3, consistent with the post-transcriptional polyadenylation model. Across all PCGs, 3798 codons (excluding stops) were identified, with leucine, serine, and isoleucine being the most frequently used amino acids, reflecting the bias toward A/T-ending codons. GC content varied moderately among genes, ranging from ~44% in ATP8 to ~52% in ND4L, suggesting differences in evolutionary constraints and substitution patterns.

### 3.2. Codon Usage and Synonymous Codon Bias

**Figure 2:**
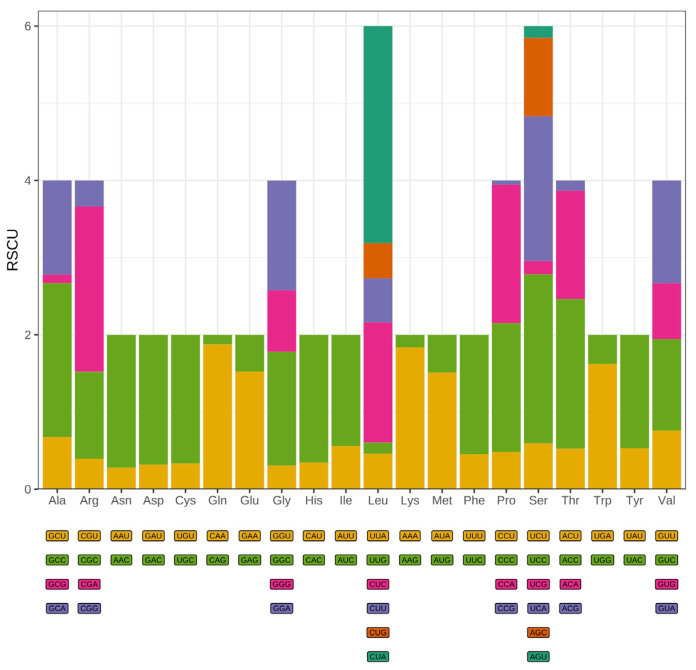
Relative synonymous codon usage (RSCU) of the *T. pallidus* mitogenome, with amino acid frequencies, the colored boxes below represent all the codons that encode each amino acid, while the height of the bars above indicates the total relative usage of synonymous codons.

The synonymous codon usage pattern of *T. pallidus* was comprehensively analyzed based on all 13 PCGs in the mitochondrial genome. A total of 3798 codons were identified across all PCGs (excluding termination codons), with individual gene lengths ranging from 56 codons (ATP8) to 606 codons (ND5) (Table S1). Consistent with patterns observed in other avian mitochondrial genomes, leucine (Leu), serine (Ser), and isoleucine (Ile) were the most abundantly used amino acids, collectively accounting for over 25% of all codons. RSCU analysis revealed pronounced codon usage bias, with several codons showing strong preference or avoidance patterns (Figure 2). Codons ending in A or T were consistently overrepresented (RSCU > 1.6), including UUA (Leu), AUU (Ile), and AUA (Met), reflecting the well-documented AT-richness of mitochondrial genomes. Conversely, codons terminating with G or C nucleotides, such as CUG (Leu), GGC (Gly), and UCG (Ser), were significantly underrepresented (RSCU < 0.6). This pattern indicates strong compositional constraints imposed by the AT-biased mutational environment typical of animal mitochondria, coupled with translational selection pressures favoring codons that pair with abundant mitochondrial tRNAs. The overall effective number of codons was approximately 40, indicating moderate codon usage bias typical of vertebrate mitochondrial genomes [31]. This value suggests that while codon usage is constrained, the mitogenome maintains sufficient flexibility to accommodate the diverse protein functions encoded by the 13 PCGs. Analysis of nucleotide composition across codon positions revealed the characteristic pattern of vertebrate mitochondrial genomes. The first codon position exhibited the highest GC content at 52.66%, reflecting the strong functional constraints on amino acid identity imposed by protein structure and function. The second codon position showed intermediate GC content (41.60%), while the third position displayed 50.29% GC content.

### 3.3. Characteristics and Secondary Structures of Mitochondrial tRNA Genes

The mitochondrial genome of the pale thrush (*T. pallidus*) contains 22 tRNA genes, ranging in length from 66 bp (shortest) to 75 bp (longest), with an average of ~70 bp and a combined length of 1543 bp, accounting for ~9.2% of the entire genome (16,739 bp) (Table S1). These tRNA genes are responsible for transporting 20 amino acids in mitochondrial protein synthesis. Comparative analysis with other avian mitochondrial tRNAs revealed that the sequences are highly conserved, with nucleotide variation mainly in the DHU arm and TψC loop, whereas the stem regions are relatively stable.

**Figure 3:**
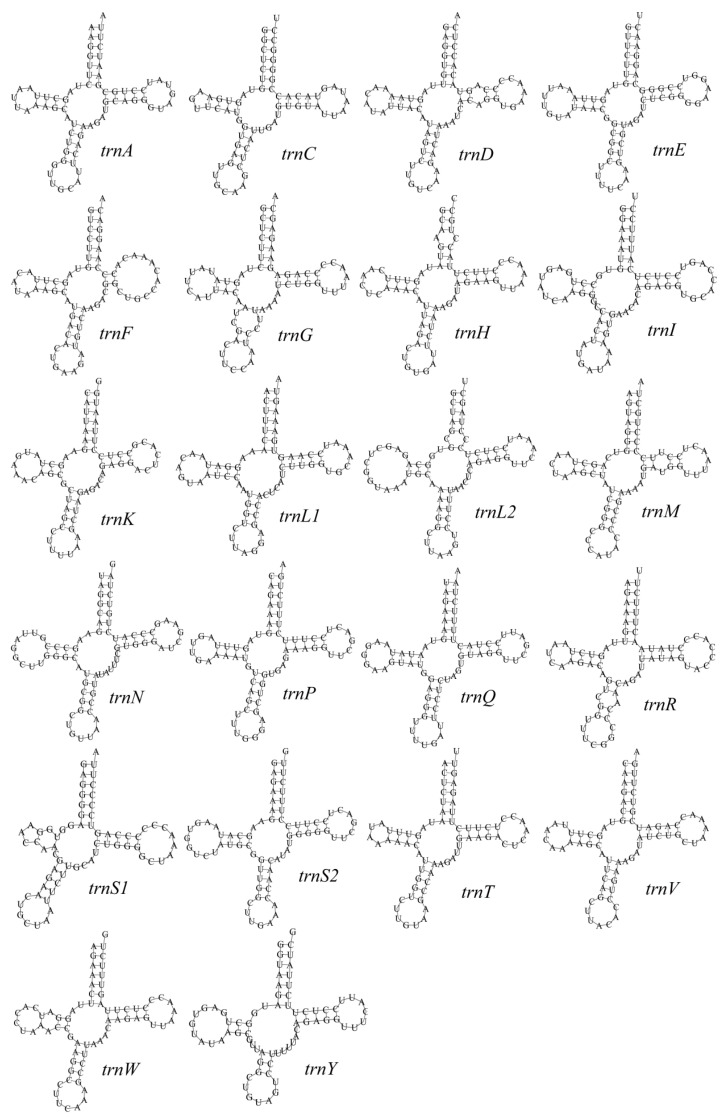
The secondary structure of tRNA gene in the mitochondrial genome of *T. pallidus.*

The tRNA genes are asymmetrically distributed: 8 genes are located on the L-strand (trnA, trnC, trnE, trnN, trnP, trnQ, trnS2, trnY) and 14 genes on the H-strand, reflecting the typical strand bias in vertebrate mitogenomes [32]. Secondary structure predictions showed that all tRNAs fold into canonical cloverleaf structures except trnS1 (AGN) (Figure 3), which lacks the DHU arm and forms a “non-canonical cloverleaf.” Standard A–U and G–C pairings dominate, while G–U wobble pairings are also common in stem regions, stabilizing the structures. Occasional single-base bulges or mismatches were observed but did not disrupt the overall topology.

### 3.4. Characteristics and Secondary Structures of Mitochondrial rRNA Genes

The mitochondrial genome of *T. pallidus* contains two rRNA genes: 12S rRNA (977 bp) and 16S rRNA (1590 bp) (Table S1), located between tRNA-Phe and tRNA-Leu, with tRNA-Val separating them. This arrangement is conserved across most avian mitogenomes. The GC contents of the two rRNA genes are 48.52% (12S) and 44.65% (16S). These results suggest that rRNA genes are under selective constraints on nucleotide composition to maintain structural and functional stability.

**Figure 4:**
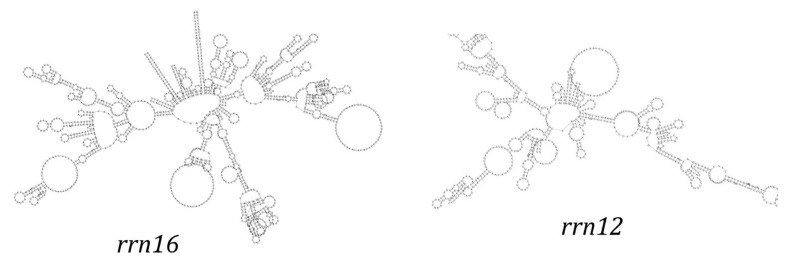
The secondary structure of rRNA gene in the mitochondrial genome of *T. pallidus.*

Secondary structure predictions indicated that both 12S rRNA and 16S rRNA fold into typical multi-domain secondary structures composed of helices and loops (Figure 4). Conserved regions were concentrated in the stem domains with strong G–C and A–U pairing, while variable regions were found in loops and bulges. In particular, the large loop regions of 16S rRNA showed greater sequence divergence, suggesting a degree of evolutionary plasticity.

### 3.5. Phylogenetic Relationships of T. pallidus

**Figure 5:**
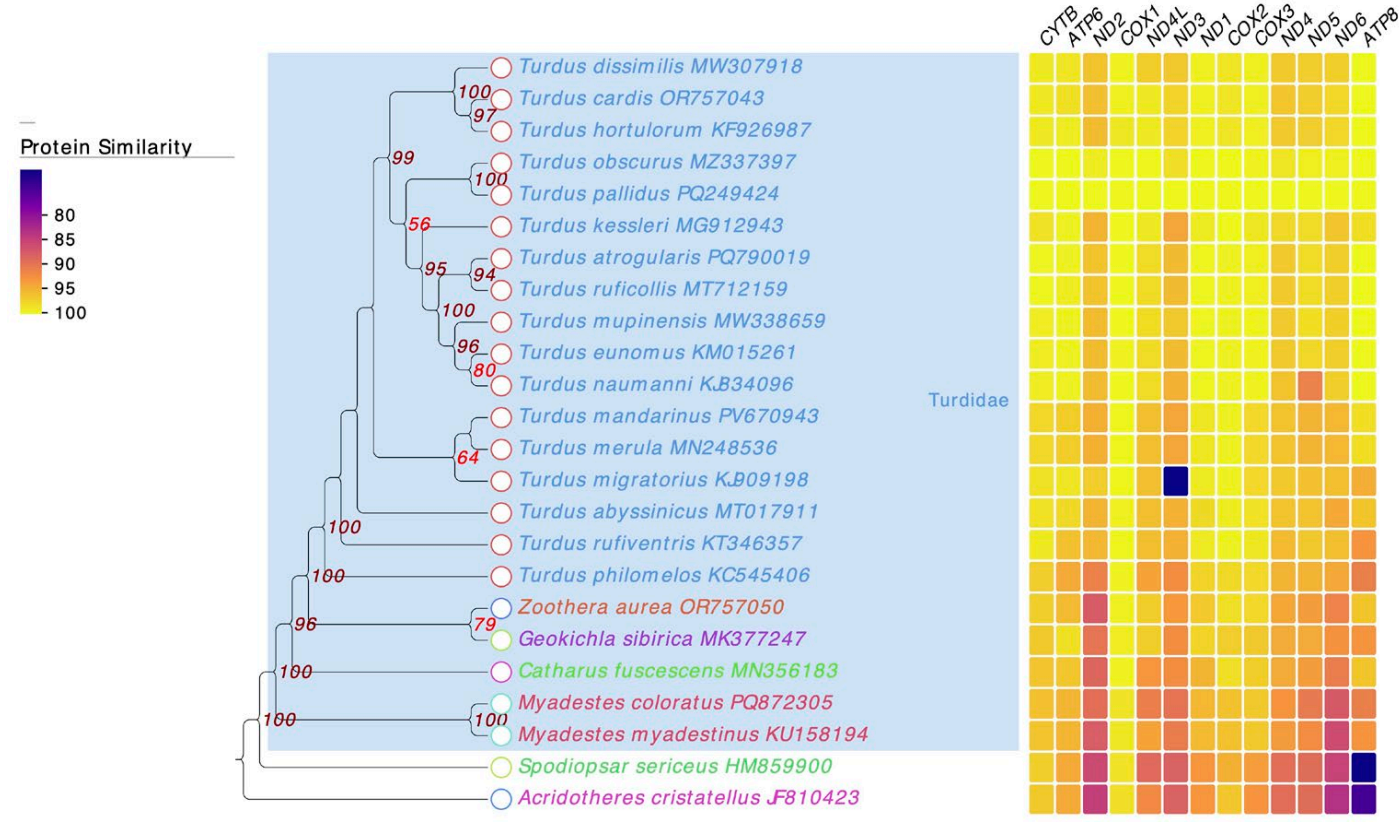
Maximum-likelihood (ML) phylogenetic tree of Turdidae species and heatmap of protein identity across mitochondrial genes, showing sequence similarity with color gradients from yellow (high identity) to purple (low identity). The numbers at the nodes represent bootstrap support values from the maximum likelihood analysis.

The phylogenetic analysis based on 13 mitochondrial protein-coding genes strongly supported the monophyly of Turdidae, with all *Turdus* species clustering together (Figure 5). Within this clade, *T. pallidus* was most closely related to *T. obscurus*. The heatmap in Figure 5 displays the percent amino-acid identity of each mitochondrial protein-coding gene in all species relative to *T. pallidus*, which was used as the reference genome for the analysis. The color scale represents varying levels of sequence conservation across the analyzed *Turdus* species. The protein identity heatmap was congruent with the phylogeny, showing high sequence conservation across *Turdus*, especially in COX1, COX2, COX3, and CYTB, while relatively variable genes such as ATP8 and ND6 exhibited lower identity across genera. Notably, outliers with lower identity values were observed for ND3 in *T. migratorius*. which often show higher variability across taxa. These patterns indicate that conserved genes are more suitable for deeper phylogenetic inference, whereas rapidly evolving genes provide greater resolution among closely related taxa. Together, these results robustly confirm the placement of *T. pallidus* within *Turdus* and clarify its evolutionary affinity with East Asian congeners (*T. obscurus* and *T. kessleri*).

## 4. Discussion

The mitochondrial genome of the pale thrush (*T. pallidus*) is highly consistent in structure and composition with other *Turdus* species. Its genome length is 16,739 bp, containing the typical set of 37 genes (13 protein-coding genes, 22 tRNA genes, and 2 rRNA genes). This result is fully consistent with reports for *T. merula* (16,730 bp), *T. naumanni* (16,750 bp), and *T. dissimilis* (16,761 bp), indicating that *Turdus* has maintained a highly conserved genomic architecture during evolution [33,34,35], as well as previously submitted mitochondrial genomes of *T. pallidus* (accession numbers OR493961 and OR493962), which show over 99.7% sequence identity with our newly generated genome (PQ249424). These findings indicate that *Turdus* has maintained a highly conserved genomic architecture during evolution.

In particular, the ND6 gene and a subset of tRNAs are encoded on the L-strand, while all other genes are encoded on the H-strand, a strand bias commonly found in vertebrate mitochondrial genomes and thought to be closely associated with asymmetric replication and transcription processes [36]. Regarding start and stop codon usage, the findings for *T. pallidus* agree with previous studies: most genes initiate with ATG, but COX1 and ND2 exhibit non-canonical start codons (GTG), a phenomenon also reported in other *Turdus* and avian species [33,34,35,37,38]. Incomplete stop codons (e.g., T– or TA–) were also identified, which rely on post-transcriptional polyadenylation to generate complete termination signals, a phenomenon widely documented in mammals and birds [39,40,41]. Thus, the start and stop codon patterns of *T. pallidus* not only reflect the compact nature of the mitochondrial genome but also align with the widespread post-transcriptional regulatory mechanisms in vertebrates.

The structural characteristics of the tRNA genes further reinforce this pattern. *T. pallidus* possesses the typical 22 tRNA genes, with lengths ranging from 66–75 bp and a total length of 1543 bp (~9.2% of the genome). All fold into canonical cloverleaf secondary structures except trnS(AGN), which lacks a DHU arm and exhibits a “non-canonical cloverleaf.” This result is consistent with observations in other birds and invertebrates [42,43], suggesting that the unusual conformation of tRNA-Ser is a recurrent structural simplification during evolution, while G•U wobble pairings play an important role in stabilizing secondary structures. The two rRNA genes (12S and 16S) of *T. pallidus* also exhibit conserved lengths and positions comparable to other *Turdus* species (12S = 977 bp, 48.52% GC; 16S = 1590 bp, 44.65% GC). Stem regions are highly conserved, whereas loop and bulge regions display greater variability, consistent with previous comparative analyses [33,37], reflecting a balance between functional stability and evolutionary flexibility.

In terms of codon usage, *T. pallidus* shows a marked preference for synonymous codons ending in A/T, such as UUA (Leu), AUU (Ile), and AUA (Met), while codons ending in G/C are underrepresented. This pattern is consistent with the general trends in avian and other vertebrate mitochondrial genomes [44,45,46]. The effective number of codons (ENC ≈ 40) indicates a moderate level of codon usage bias, reflecting the combined effects of mutational pressure and selection for translational efficiency. The GC content at codon positions shows that the first position is richest in GC (52.66%), followed by the second (41.60%) and third (50.29%) positions, further illustrating a balance between mutation pressure and selection. These findings align with previously reported patterns of codon bias in birds [47].

Phylogenetic analysis strongly confirmed the monophyly of Turdidae, with all *Turdus* species clustering together. Within the genus, *T. pallidus* was most closely related to *T. obscurus*. This finding is consistent with earlier studies based on mitochondrial or nuclear genes [1,10,30,48], but the use of complete mitogenomic data in this study provided stronger support. In conclusion, the mitochondrial genome of *T. pallidus* not only provides a novel genomic resource for molecular species identification but also offers robust evidence for clarifying phylogenetic relationships and evolutionary history within *Turdus*. Compared with previous studies, the present work improves both data completeness and phylogenetic resolution, underscoring the value of complete mitogenomes in species identification, conservation management, and avian evolutionary studies.

## Data Availability

The data that support the findings of this study are available in the National Center for Biotechnology Information (NCBI) at Genbank with accession number PQ249424 (https://www.ncbi.nlm.nih.gov/nuccore/PQ249424.1 (accessed on 30 September 2025)).

## Supplementary Materials

The following supporting information can be downloaded at: https://www.mdpi.com/article/10.3390/genes16111284/s1, Table S1: Characteristics of the complete mitochondrion genome of *T. pallidus*; Table S2: Mitochondrial Genome Statistics of *Turdus* and related species.
